# The importance of the medical imaging technology park: access, equity, and balance

**DOI:** 10.1590/0100-3984.2024.57.e2-en

**Published:** 2024-05-31

**Authors:** Claudia Marques Canabrava, Altacílio Aparecido Nunes, Antônio Pazin Filho

**Affiliations:** 1 Postdoctoral student, Faculdade de Medicina de Ribeirão Preto da Universidade de São Paulo (FMRP-USP), Ribeirão Preto, SP, Brazil. Email: claudia.canabrava@usp.br; 2 Tenured Full Professor, Department of Medicine, Universidade Federal de São João del Rei (UFSJ), São João del Rei, MG, Brazil. Email: altacilio@ufsj.edu.br; 3 Full Professor of Emergency Medicine, Faculdade de Medicina de Ribeirão Preto da Universidade de São Paulo (FMRP-USP), Ribeirão Preto, SP, Brazil. Email: apazin@fmrp.usp.br

It is essential to consider the need for accurate and rapid diagnoses, especially in cases of cancer and other serious illnesses, including those that are rare or ultra-rare. Planning and programming care based on population-based parameters and loco-regional distribution (travel time and distance) can guarantee quick, equitable access to technologies that enable therapeutic success or mitigation of sequelae^(1)^. Therefore, medical imaging has become a fundamental element of health care, playing an unquestionable role in all stages of health care provision^(2)^, from prevention to monitoring and treatment follow-up.

In a recent issue of **Radiologia Brasileira**, Alencar et al.^(3)^ provide an interesting descriptive evaluation of a time series on the geographical distribution of devices, notably computed tomography (CT) and magnetic resonance imaging (MRI) scanners—equipment of high technological density—and on the performance of highly complex radiological procedures, comparing the regions of Brazil, as well as comparing the public and private sectors, over a period of seven years (2015–2021).

The results demonstrate disparity in all variables studied (from the distribution of equipment to the number of procedures) between the regions of Brazil, as well as between the public and private health care sectors in Brazil. One interesting finding is that the total number of MRIs performed in the private sector in 2021 (7,834,285) exceeded the total number expected for the entire population of Brazil in the same period, based on the parameter of 30 procedures per year per 1,000 inhabitants established by Ordinance GM/MS 1631/2015^(4)^. Identifying the population for which the examination was carried out—individuals receiving “supplementary health care” (covered by a private insurance plan) or those receiving care via the *Sistema Único de Saúde* (SUS, Unified Health Care System)—and stratifying the examination site (private vs. public) will support more refined analyses, both of the utilization rate (number of exams per population group) and of the participation of the private sector in the use of procedures of high technological complexity covered by the SUS (i.e., the number of private establishments offering diagnostic support services to the SUS).

Souza et al.^(5)^ reported that, in 2012 in Brazil, most (92.46%) of the facilities exclusively providing diagnostic support and treatment were private. In addition, the same authors highlighted that, despite the considerable growth in the absolute number of devices in the 2008–2022 period (from 747,000 to 4,857,000), their *availability for use via the SUS in 2023 did not keep pace with that*, only 30% of the existing total number diagnostic imaging devices being used via the SUS in 2023^(6)^.

The overall inequality in access to health care services between the public and private sectors and among the regions of Brazil is considerable and has widened over the years. During the coronavirus pandemic, the major expansion in access to ICU beds in the public sector (66% growth) was not sufficient to mitigate that inequity, and a third of the regions of the country had ICU beds available only for the population of individuals with private health insurance, who were 3.7 times more likely to have access to this type of resource than were those receiving health care via the SUS^(7)^.

The Brazilian National Ministry of Health recently established, within the scope of the SUS, the National Program for the Expansion and Qualification of Specialized Outpatient Care^(8)^, which aims to expand access to consultations and examinations, as well as to other diagnostic and therapeutic procedures. The program proposes that the organization of health measures and services should be based on the provision of integrated care, which, in turn, should be based on care parameters. Therefore, studies like that conducted by Alencar et al.^(3)^ support the analysis and identification of opportunities to expand access and reduce avoidable inequalities in the right to specialized diagnostic imaging procedures.
